# Supporting pregnant women not ready to quit smoking: an economic evaluation

**DOI:** 10.1186/s12884-022-05150-8

**Published:** 2022-11-23

**Authors:** Tuba Saygın Avşar, Louise Jackson, Pelham Barton, Matthew Jones, Hugh McLeod

**Affiliations:** 1 Department of Applied Health Research, NIHR ARC North Thames and UCLPartners Academic Health Science Partnership, University College London, London, WC1E 7HB UK; 2 Health Economics Unit, University of Birmingham, Birmingham, UK; 3 Division of Primary Care, University of Nottingham, Nottingham, UK; 4 Population Health Sciences, Bristol Medical School, University of Bristol, Bristol, UK; 5 NIHR ARC West at University Hospitals Bristol and Weston NHS Foundation Trust, Bristol, UK

**Keywords:** Smoking cessation, Tobacco, Pregnancy, Economic evaluation, Cost-effectiveness, Reduction, Financial incentives

## Abstract

**Objectives:**

Some pregnant women are not ready or do not want to quit smoking completely, and currently there is no support provided for these women in the UK. Offering help to reduce smoking could reduce the health risks associated with smoking and increase the limited reach of the NHS Stop Smoking Services (SSS) for pregnant women. This study aimed to design and evaluate a hypothetical intervention aimed at pregnant women who are not yet ready or do not want to quit smoking entirely.

**Methods:**

A hypothetical intervention, the Reduced Smoking During Pregnancy (RSDP) intervention, was conceptualised based on the best available evidence. The intervention was evaluated, using a decision-analytic model developed for SDP interventions. Two different scenarios, a base-case and a cautious-case were developed, and a cost-utility analysis and return on investment analysis were conducted. The uncertainty around the estimates was assessed, using deterministic and probabilistic sensitivity analyses.

**Results:**

The RSDP intervention could prevent the loss of 13 foetuses and generate 43 quitters 1 year after delivery per 1000 women. In the lifetime analysis, the intervention was cost-effective in both scenarios, with an incremental cost of £363 (95% CI £29 to £672) and 0.44 (95% CI 0.32 to 0.53) QALYs gained in the base-case.

**Conclusions:**

The study found that the hypothetical reduction intervention would produce significant health benefits, reduce smoking and be cost-effective. Offering pregnant smokers help to reduce smoking could reduce health inequalities, widen the reach of SSS and improve health. This economic evaluation of a novel, intensive intervention could inform the piloting of such interventions.

**Supplementary Information:**

The online version contains supplementary material available at 10.1186/s12884-022-05150-8.

## Introduction

Many women quit smoking once they realise that they are pregnant due to the significant health risks associated with smoking during pregnancy (SDP) [1, 2]. However, around half of pregnant women who smoke do not quit during their pregnancy in the United Kingdom (UK) [3]. Women from lower socioeconomic backgrounds and those who live with other smokers are less likely to quit during pregnancy; therefore, SDP is a health inequality issue [4]. Currently, in the UK, there is no support for pregnant women who wish to reduce their smoking but are not ready to quit. This partly explains the limited reach of NHS Stop Smoking Services (SSS), given that only around 12% of women who smoke during pregnancy engage with SSS [1, 5]. A potential way to increase the reach of such services is to include those who are not ready to quit, by offering help with reducing the number of cigarettes consumed, which lowers the health risks for the mother and infant significantly [6–12]. Additionally, women who smoke fewer cigarettes are more likely to quit smoking later in life and are less likely to expose their children to second-hand smoking [13–18].

The interventions designed for women who smoke during pregnancy have usually targeted women who wanted to quit and included only those who were ready to quit. There were some trials which aimed to help women quit gradually by reducing the number of cigarettes consumed [19]. However, there is a lack of evidence on the cost-effectiveness of interventions which aim to support women who want to reduce their consumption but are not yet ready to quit completely.

A recent systematic review of SDP interventions identified six interventions that helped pregnant women, who wanted to quit eventually, reduce their daily cigarette consumption [19–25]. Of these, two did not provide a clear definition of reduction [21, 23] and one was published as an abstract which did not contain the intervention details [20]. Two trials involved educational materials and behavioural support and reported a 5% increase in the proportion of participants who reduced smoking by 50% or more during late pregnancy [24, 25]. The final intervention [22] involved financial incentives and reported that 48% of women in the intervention group who were provided with contingent financial incentives achieved a 75% reduction in smoking compared with none in the control group. Hence, the evidence shows that behavioural support and financial incentives could help pregnant smokers to reduce the number of cigarettes consumed. However, there is no published study on the effectiveness of combining behavioural support with high levels of financial incentives to assist pregnant women who would like to reduce their smoking but do not want to quit entirely. Thus, this study aimed to explore the potential health and cost implications of such an intervention.

In the absence of evidence on the effectiveness and cost-effectiveness of interventions to reduce smoking in pregnant women who do not want to quit, this study conceptualised a hypothetical intervention for pregnant women who want to reduce their smoking consumption (but are not yet ready to quit entirely) and estimate its cost-effectiveness. To the best of the authors’ knowledge this is the first economic evaluation of an intervention aimed to help support women who would like to reduce the amount of smoking during pregnancy. The findings could inform decisionmakers in designing appropriate services for pregnant women in the UK, and potentially in other countries.

## Methods

### Population, intervention and control

The target population was pregnant women in the UK who smoke and want to reduce their smoking consumption although they are not committed to quitting. The thresholds to define light, moderate and heavy smokers were based on a systematic review, as follows: 1–10, 11–20, and 21 or more cigarettes daily, respectively [2].

The hypothetical intervention was developed by combining elements of published studies [22, 24, 25]. The key features of the hypothetical *Reduced smoking during pregnancy (RSDP) intervention* were an initial counselling session in a maternity care setting, a leaflet, financial incentives and home visits. Financial incentives have been found to be the most effective and cost-effective intervention for quitting smoking during pregnancy, and effective in supporting pregnant women to reduce smoking consumption [19, 22, 26]. Hence, the RSDP intervention was designed to include monthly shopping vouchers against negative specimens in addition to behavioural support. In a discrete choice experiment in the UK, pregnant smokers reported that receiving monthly vouchers with a value of £40 and higher would increase their probability of quitting [27]. Thus, the amount of shopping vouchers in the RSDP intervention was defined as £40 per specimen which showed reduction in carbon monoxide levels.

In England, half of the first maternal care appointments took place between 7th and 12th weeks of gestation while the other half occurred by 6 weeks [28]. Thus, it would be reasonable to assume that the first appointment would take place during the seventh week. Based on evidence showing that the most critical times for behavioural change is the first few weeks of pregnancy and around the time of delivery, weekly home visits were planned for the first 4 weeks after the initial meeting and during the 4 weeks just before the delivery, with monthly visits in between [27, 29]. This would result in 12 home visits by the point of delivery, in addition to the initial contact. The women in the control group would not receive any specific support to reduce smoking, as per usual practice in the UK.

### Effectiveness

The effectiveness of the intervention was measured with the number of women who reduce smoking at delivery and the impacts of this on mothers, partners and the offspring at different time points (e.g. 1 year after delivery and lifetime) were captured. Due to the absence of a trial focusing on this specific group of women, a base-case and a cautious-case were designed based on the available evidence. The trial by Tuten et al. [22] which included methadone maintained women was utilised to estimate the expected effectiveness of the hypothetical RSDP intervention since this was the only trial assessing the effectiveness of financial incentives to reduce smoking in pregnant women although they included pregnant women who wanted to quit eventually. In this trial, women were awarded up to $41.5 over 12 weeks, contingent on setting a quit date and reducing their cigarette consumption by 75% or more. Reduction in smoking was confirmed with a carbon monoxide (CO) level less than 4 ppm and cotinine testing lower than 200 ng/ml. In this trial, 48% of the participants reduced smoking by 75% or more. In addition, a meta-analysis of interventions targeting substance use [30], including smoking, estimated that providing above $16 daily would increase the effect by 43%. Therefore, it was reasonable to assume that the RSDP intervention would be more effective than the intervention by Tuten et al. [22] due to the significantly greater amount of incentives offered per woman, and also the increased number of contacts [31]. Hence, in the base-case, it was assumed that 69% of women would reduce smoking by 75%, based on a 43% greater impact than the trial by Tuten et al. [22].

The proportions of light, moderate and heavy smokers in the control and intervention groups are provided in Table 1. It was assumed that women in the control group would not reduce the number of cigarettes consumed, and the proportions of light, moderate and heavy smokers at delivery were assumed to be 0.40, 0.32, and 0.28 respectively, based on a recent UK trial [32]. Since the intervention was designed for expectant mothers who were not ready to quit, it was assumed that no women would quit by delivery although they would reduce the number of cigarettes consumed. Reduction was defined as reducing cigarette consumption by 75%, and a reduction in smoking would not change the categorisation of those defined as light smokers at baseline, because they would be considered as light smokers even if they smoked only one per day. Thus, the proportions in the base-case would be 0.81, 0.10 and 0.09 for light, moderate and heavy smokers respectively. In the cautious-case, it was assumed that 48% of women would reduce smoking by 75% based on the effectiveness level reported by Tuten et al. [22], and the proportions of light moderate and heavy smokers would be 0.69, 0.17 and 0.14 respectively.

**Table 1:** Smoking groups in the control group and in the hypothetical RSDP intervention (at delivery)

Scenarios	Outcomes	Of smokers	Based on
Control group	Light smokers	0.40	Cooper et al. 2017 [32]
	Moderate smokers	0.32	
	Heavy smokers	0.28	
Base-case	Light smokers	0.81	Tuten et al. 2012 [22], Lussier et al. 2006 [30]
	Moderate smokers	0.10	
	Heavy smokers	0.09	
Cautious-case	Light smokers	0.69	Tuten et al. 2012 [22]
	Moderate smokers	0.17	
	Heavy smokers	0.14	

Based on the SDP trials and national datasets, it was assumed that 68% of pregnant smokers had smoking partners during pregnancy and women with smoking partners were less likely to quit smoking during pregnancy [3, 32]. However, in the absence of data on the relationship between reducing smoking during pregnancy and partner’s smoking, it was assumed that it did not have any impact on the effectiveness of the RSDP intervention. It was also assumed that the probability of quitting smoking 1 year after delivery was the same for the intervention and control groups. However, the RSDP intervention would increase the number of quitters 1 year after delivery because light and moderate smokers were more likely to quit smoking than heavy smokers.

### Costs

All costs were reported in 2017 prices in order to facilitate comparison with other analysis based on the ESIP.H model, which is described below [33]. The unit costs that were used to estimate the intervention were derived from the published literature and NHS Reference Costs (Additional File, Table A1). Since the financial incentives would be provided contingent on biochemical verification, it was assumed that the actual voucher costs per women would be lower than the total maximum amount available (£520). The expected cost of vouchers was estimated by multiplying the expected proportion to reduce smoking by the cost of each voucher (£40) and the postage cost (£1.8). Thus, the estimated cost of vouchers per woman were £386 in the base-case and £281 in the cautious-case. Similarly, the costs of midwife visits and CO monitoring was estimated based on the proportion expected to reduce smoking, and the estimated costs for this were £470 in the base-case and £327 in the cautious-case.

### Analysis and outcomes

The study was a literature-based economic modelling analysis of a hypothetical complex intervention to inform intervention development, which was not pre-registered [34]. Cost-effectiveness is an important consideration for optimal decision-making in healthcare [35]. Cost-effectiveness of healthcare interventions is assessed by estimating the additional costs and health benefits of a novel intervention compared to existing services [36]. The health benefits of smoking cessation interventions can be measured in terms of the number of quitters or those who reduce consumption, expected life years (LYs) and quality-adjusted life years (QALYs) [37]. The studies using QALYs are defined as cost-utility analyses since utility gain expected from an intervention is considered by estimating QALYs along with additional costs [38]. According to the National Institute for Care and Excellence (NICE) in the UK, any public health intervention which generates an additional QALY for a cost between £20,000 and £30,000 is considered cost-effective [35].

Additionally, to estimate the monetary benefits of healthcare interventions for return on investment (ROI) analyses are conducted [39]. ROI is obtained by dividing the additional benefits by the additional costs, which provides the expected monetary gain for each £1 invested. ROI enables decision makers compare healthcare services with other types of services, such as social care. ROI is usually calculated in two different ways: one covering the NHS healthcare cost savings only (ROI1) and the second capturing health gains as well (ROI2), by assigning a monetary value of £30,000 per QALY gain.

In this study, a cost-utility analysis and ROI analysis were conducted from an NHS perspective hence only the costs to the healthcare funder were included, and a discount rate of 3.5% was applied to both costs and benefits. The analyses were conducted for a base-case and a cautious-case. The main outcomes were the number of quitters 1 year after delivery and incremental QALY gains and incremental costs per woman reducing their smoking. The outcomes were estimated separately for mother and offspring, combined for mother and offspring, and combined for household (mother, offspring, and mother’s partner). Different time horizons were applied such as 1 year after delivery and lifetime since reducing smoking has short-term and long-term impacts.

A deterministic sensitivity analysis was undertaken to explore the maximum value for cost per woman and the minimum value for the proportion of women who reduce smoking at which the RSDP intervention would remain cost-effective. The analysis was also repeated, assuming women who were exposed to second-hand smoking had a 50% less probability of reducing smoking, reducing the postpartum quit rates amongst mothers by 50% and applying a discount rate of 1.5% as per NICE guidelines [35]. A probabilistic sensitivity analysis (PSA) was conducted to analyse the uncertainties around the model findings and to explore the probability of cost-effectiveness at different thresholds, and cost-effectiveness acceptability curves (CEACs) were produced. All the analyses were conducted in Microsoft Excel.

### The ESIP.H model

**Fig. 1:**
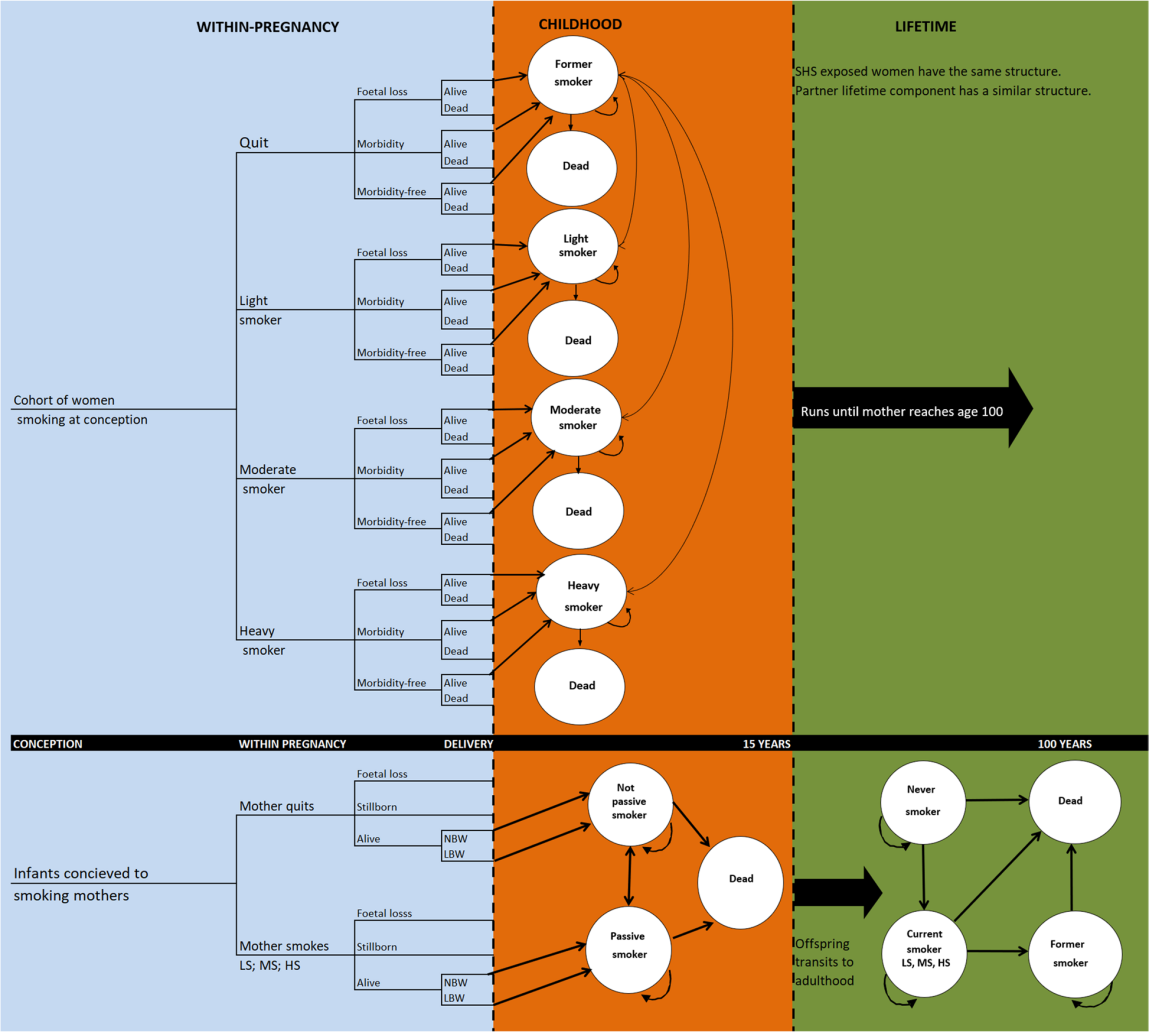
Simplified illustration of the ESIP.H model

A decision-analytic model called the “Evaluation of Smoking in Pregnancy-Household (ESIP.H)” model was utilised to conduct the cost-utility and ROI analyses, which is described in full elsewhere [33]. The structure of ESIP.H is provided in Fig. 1. In brief, the model is a dynamic Markov model which estimates the additional health benefits and costs expected from smoking cessation and smoking reduction interventions during pregnancy. The model consists of a decision tree for the pregnancy period and four linked Markov chains for mother lifetime, partner lifetime, offspring childhood (until age 15) and offspring adulthood (age 16 and over). The model groups pregnant women based on their second-hand smoke exposure by partners, and then into four groups as former, light, moderate and heavy smokers. The health conditions considered in the model were identified based on a systematic review [2]. It was assumed that SHS during pregnancy had no additional impact on active smokers because no study was identified showing this relationship [12].

The model starts with 1000 pregnant smokers and their partners and runs in annual cycles throughout the lifetime of the offspring. The user enters the average age of mothers and partners and the birth year since the model uses specific mortality risks based on this. These were set to be 27 (same for mothers and partners) and 2017 in this study. The user enters the probability of quitting smoking amongst pregnant women at the time of delivery and 1 year after delivery. For this study, it was assumed that no women would quit at the time of delivery; but some would quit 1 year after delivery. The user can also enter the proportions of light, moderate and heavy smokers into the model for both control and intervention arms. Similarly, the proportion of pregnant women who are exposed to second-hand smoke and the probability that smoking partners would quit can be entered into the model. In this study, it was assumed that the proportion exposed to second-hand smoke was the same both in the control and intervention (65%) [3, 32], and none of the smoking partners would quit at the time of delivery. In addition, the probability of second-hand smoke exposure amongst children was 65% if mothers were light or moderate smokers and 93% if they were heavy smokers [33].

ESIP.H was used for the current analysis as it is the only available model which considers the severity of smoking and mothers’ partners’ smoking in addition to the mothers’ smoking. The smoking status of partners impacted on the probability of quitting amongst mothers starting from the first year after delivery. Moreover, the smoking status of mothers and mothers’ partners affected second-hand smoking rates amongst the children and the smoking uptake at the age of 15. Due to the absence of data, it was assumed that the number of cigarettes consumed would not change after entering the model at the time of delivery, unless the individuals quit and then restarted smoking. Those who relapsed to smoking were distributed into light (0.46), moderate (0.40) and heavy smokers (0.14) based on the proportions in the general population [40].

## Results

The total cost of the RSDP intervention per woman was estimated at £856 in the base-case. The deterministic results showed that out of a hypothetical cohort of 1000 pregnant women who did not want to quit, 411 in the base-case would reduce cigarette consumption by 75% (Table 2). The RSDP intervention was estimated to prevent the loss of 13 foetuses and 9 low birth weights (LBW). The intervention also generated 43 additional quitters 1 year after delivery since light and moderate smokers would be more likely to quit smoking than heavy smokers. In the cautious-case, the cost of RSDP per woman was estimated to be £607 and the results for the cautious-case are provided in the Additional File (Table A3).

**Table 2:** ESIP.H outcomes for RSDP base-case

	Deterministic			Control (PSA)			Intervention (PSA)			Incremental outcomes (PSA)		
End of pregnancy	Control	Intervention	Incremental	Mean	95% CI		Mean	95% CI		Mean	95% CI	
Number of reducers	0	411	411	0	0	0	410	371	450	410	371	450
Number of infants lost	105	92	−13	105	103	108	92	91	93	−13	−15	−11
Number of infants born prematurely	70	70	0	70	69	71	70	69	70	0	0	0
Number of infants born with LBW	103	94	−9	104	102	105	95	94	95	− 9	−10	−8
Expected LYs per mother	0.71	0.71	0.0068	0.71	0.71	0.71	0.71	0.71	0.71	0.0068	0.0061	0.0080
Expected QALYs per mother	0.63	0.65	0.0139	0.63	0.62	0.64	0.65	0.63	0.66	0.0139	0.0082	0.0195
Incremental cost per mother and offspring	£7050	£7981	£932	£6279	£3358	£12,210	£7192	£4253	£13,241	£913	£734	£1097
Incremental cost per QALY			**£66,893**									**£65,813**
End of 1 year after delivery												
Number of quitters (mother)	127	169	43	127	0	0	0	0	0	0	0	0
Expected LYs per mother and infant	2.53	2.55	0.0193	2.53	2.53	2.54	2.55	2.55	2.55	0.0162	0.0129	0.0199
Expected QALYs per mother and infant	2.34	2.38	0.0393	2.34	2.30	2.37	2.38	2.34	2.41	0.0393	0.0258	0.0535
Expected cost per mother and infant	£7463	£8351	£888	£6684	£3760	£12,609	£7534	£4577	£13,566	£851	£670	£1033
Incremental cost per QALY			**£22,606**									**£21,662**
Offspring age 15 (end of childhood)												
Number of SID	1	1	0	1	1	2	10	10	10	0	0	0
Expected LYs per child	10.25	10.40	0.1492	10.25	10.21	10.27	10.40	10.38	10.41	0.1499	0.1344	0.1761
Expected QALYs per child	10.01	10.19	0.1777	10.01	9.67	10.21	10.19	9.89	10.36	0.1788	0.1461	0.2367
Expected cost per child	£7272	£7694	£422	£6989	£4461	£12,924	£7382	£4819	£13,279	£392	£74	£669
Incremental cost per additional QALY per child			**£2374**									**£2194**
Mother and offspring lifetime combined												
Expected LYs	49.23	49.60	0.3683	49.26	49.14	49.40	49.63	49.52	49.76	0.3694	0.3296	0.4329
Expected QALYs	44.06	44.50	0.4389	44.26	43.53	44.83	44.70	43.97	45.26	0.4391	0.3715	0.5271
Expected cost	£37,639	£38,126	£487	£35,778	£31,953	£42,207	£36,229	£32,467	£42,447	£451	£127	£743
Incremental cost per reducer at delivery			£0.99									£0.88
Incremental cost per QALY			**£1110**									**£1027**
Household (mother, partner, and offspring)												
Expected LYs	74.14	74.51	0.3719	74.20	74.00	74.44	74.57	74.39	74.82	0.3734	0.3334	0.4370
Expected QALYs	66.41	66.85	0.4414	66.63	65.57	67.45	67.07	66.00	67.87	0.4423	0.3740	0.5302
Expected cost	£70,932	£71,338	£406	£68,284	£62,036	£75,709	£68,647	£62,384	£76,252	£363	£29	£654
Incremental cost per QALY			**£919**									**£820**
Return on investment												
ROI1 1 year after delivery			−0.04							−0.08	−0.19	−0.02
ROI1 offspring and mother lifetime			0.43							0.08	0.03	0.13
ROI1 household lifetime			0.53							0.45	0.18	0.81
ROI2 1 year after delivery			1.33							0.46	0.26	0.70
ROI2 offspring and mother lifetime			15.82							2.73	0.74	0.74
ROI2 household lifetime			16.00							16.12	12.39	21.45

The incremental cost per QALY at delivery was significantly higher than the NICE threshold of £30,000. One year after delivery, however, the intervention was cost-effective in the base-case (£22,606) and in the cautious-case (£23,051). The combined lifetime analysis for the mother and offspring demonstrated that RSDP was cost-effective, with an incremental cost of £1110 per QALY gained in the base-case £1162 in the cautious-case. The incremental costs per QALY reduced when the impacts on the partner were also considered, with values of £919 in the base-case analysis and £970 in the cautious-case. According to the ROI analysis, the return on investment would not cover the investment if the health gains were not considered. However, when the QALY gains were also considered, the intervention would provide £16 in the base-case and £15.67 in the cautious-case for every £1 invested.

### Sensitivity analysis

**Fig. 2:**
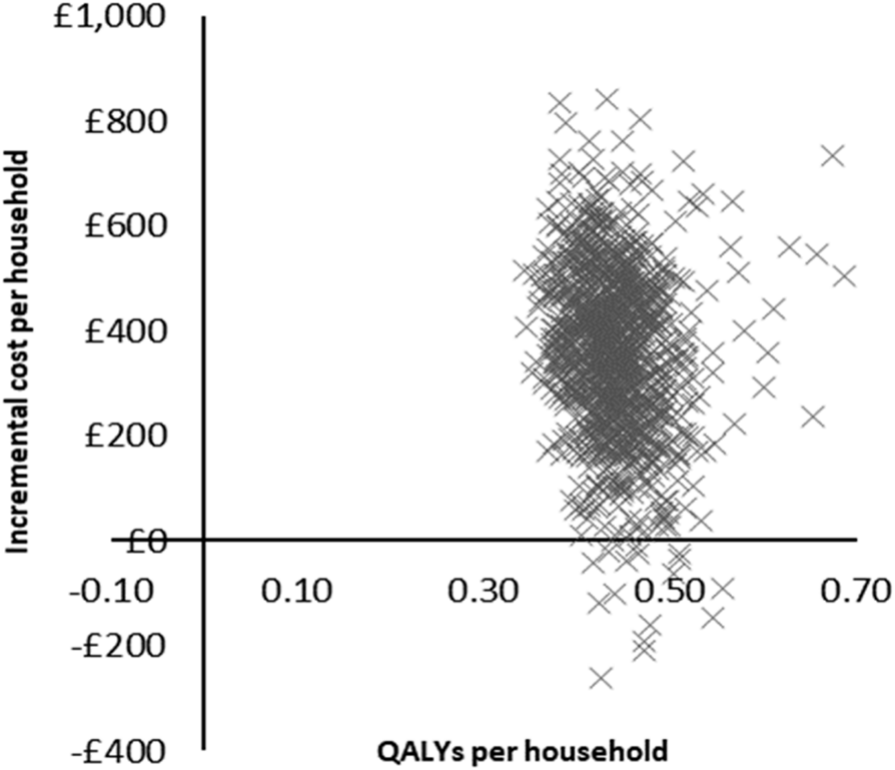
Scatterplot for base-case probabilistic sensitivity analysis (PSA)

**Fig. 3:**
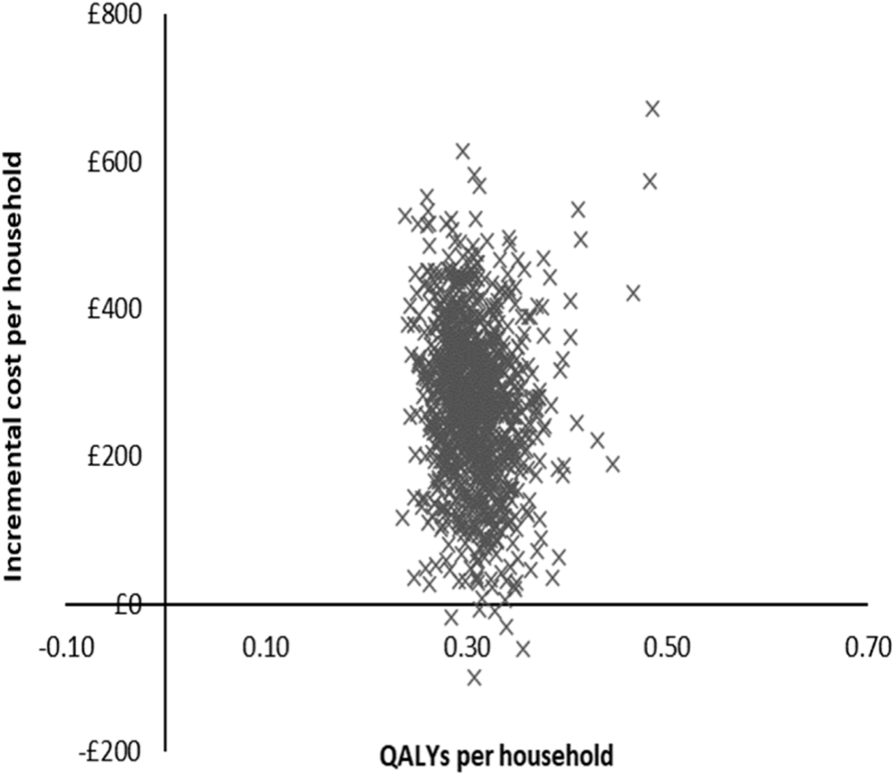
Scatterplot for cautious-case probabilistic sensitivity analysis (PSA)

**Fig. 4:**
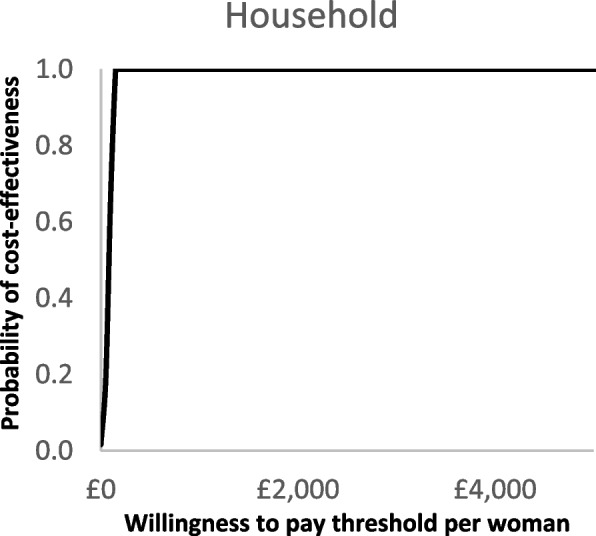
Cost-effectiveness acceptability curve (CEAC) for base-case

**Fig. 5:**
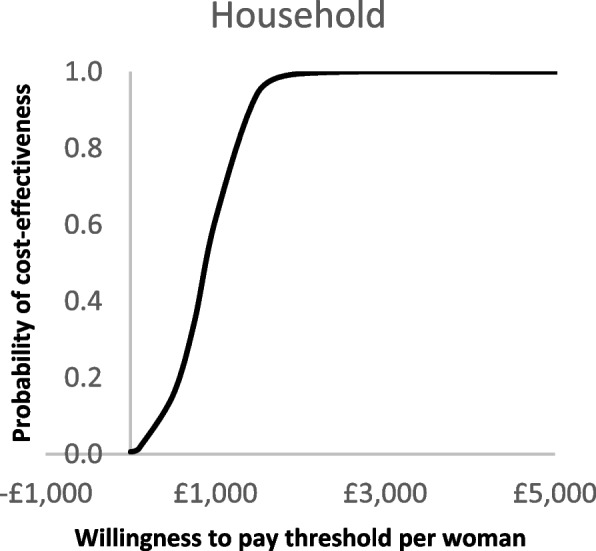
Cost-effectiveness acceptability curve (CEAC) for cautious-case

The deterministic sensitivity analysis showed that the RSDP intervention would remain highly cost-effective even when the intervention cost per woman was increased to £9350 with an ICER per QALY lower than £20,000 (Additional File, Figs. A1 and A2). Similarly, the intervention remained highly cost-effective when assuming only 6% of woman would reduce smoking at the same intervention costs. Reducing the postpartum quit rates by 50% did not have a significant impact on the outcomes while applying a discount rate of 1.5% reduced ICER per QALY to £719. In the PSA, model convergence was reached at 1000 iterations. The findings of the PSA were similar to the deterministic analysis and all the differences between the hypothetical RSDP intervention, and the control were statistically significant (Table 1). In the household lifetime analysis, the intervention was cost-effective with an incremental cost of £363 (95% CI £29 to £654) and 0.44 QALYs gained. The PSA scatterplots are provided in Figs. 2 and 3. The outcomes of the PSA for the cautious case analysis are provided in the Additional File (Table A3). The cost-effectiveness acceptability curves (CEACs) showed that, in the lifetime analysis, the probability of cost-effectiveness was 100% when the WTP per QALY was £1000 or above in both scenarios (Figs. 4 and 5).

## Discussion

This study evaluated a hypothetical intervention designed for pregnant women who want to reduce smoking but are not yet ready to quit, using the ESIP.H decision analytic model. The analyses showed that the RSDP intervention was not cost-effective at delivery but became cost-effective when the benefits beyond delivery were also considered.

### Strength and limitations

To the best of the authors’ knowledge, this is the first study to assess the cost-effectiveness of an intervention designed specifically for pregnant smokers who are not ready to quit entirely. Thus, the analysis was challenging due to the paucity of data. The only published intervention providing financial incentives for pregnant women who set a quit date and achieved reduction targets included drug-dependant women [22]. Hence, the impact of financial incentives on women who are not drug-dependant might be different. If they are more effective amongst drug-dependant women, then the study might be potentially overestimating the benefits of RSDP. In contrast, if they are less effective amongst that group, the study would be underestimating the benefits of the intervention. Also, in the absence of data, it was assumed that having a smoking partner during pregnancy would not make a difference on the probability of reducing cigarette consumption in the base-case. This was addressed in the deterministic sensitivity analysis, entering lower reduction rates for those who were exposed to second-hand smoking. Should new evidence become available this can be incorporated into the ESIP.H model. Another consideration is that there is limited evidence on the feasibility and acceptability of such interventions for pregnant women who are not yet ready to quit. Hence, further research on the feasibility and acceptability of the RSDP intervention is needed.

The ESIP.H model used in this study is the most comprehensive economic model developed for assessing the interventions aimed at pregnant women who smoke, and it is the only model to consider the severity of smoking and partner smoking. However, limitations arising from modelling assumptions should be considered. For example, in the absence of data regarding the impact of change in the number of cigarettes consumed daily during pregnancy, it was assumed that women would not change their smoking behaviour between conception and delivery, and the implications of this assumption on model outcomes are unknown. Similarly, it was assumed that the severity of smoking would not change after entering the model unless a smoker quit and then re-started smoking. The impact of this on the findings is unknown since there is no published evidence on how the number of cigarettes consumed changes over the long term. Additionally, the generalisability of health economics findings to other settings is limited since ESIP.H used UK-based data. Nonetheless, the characteristics of optimum cessation interventions might be relevant to many other countries, with similar healthcare systems [41]. Hence, the study may be repeated for different countries after re-parameterising the ESIP.H model.

### Implications

SDP has reduced significantly to 10% in the UK. However, achieving the national target of 6% by 2023 is not possible with the existing services. The study proposes a novel intervention to support pregnant women who are not yet ready to quit smoking and shows that this might help 43 women quit smoking until 1 year after delivery, producing significant health benefits. Given the budget constraints, a major concern for decision-makers would be the increased costs of providing such interventions. According to the economic evaluation, such interventions have the potential to be highly cost-effective with a cost up to £9350 per woman. Considering high SDP rates amongst women from low socioeconomic backgrounds in many countries, these interventions could help reduce the gap in health inequalities by supporting deprived households. Furthermore, the study demonstrates how the ESIP.H model can be used to design and evaluate similar interventions for pregnant women who are not committed to quitting smoking.

## Conclusion

By offering support to those not committed to quitting, healthcare providers can increase the reach of their services as well as achieving greater health benefits. The findings suggest that interventions aiming to reduce cigarette consumption during pregnancy warrant attention, and thus support for women who are not ready to give up smoking should be considered for SDP programmes. In conclusion, the economic analysis shows that, providing intensive behavioural support alongside comparatively high levels of financial incentives for pregnant women to reduce smoking consumption is very likely to be cost-effective and that piloting the RSDP intervention could be relevant for health policymakers in the UK.

## Supplementary Information

**Additional file 1.**

## Data Availability

All the data used in this study are from published studies or national datasets, which available online. https://pubmed.ncbi.nlm.nih.gov/22716774/ https://pubmed.ncbi.nlm.nih.gov/8427323/ https://pubmed.ncbi.nlm.nih.gov/23483412/ https://www.cqc.org.uk/sites/default/files/20200128_mat19_statisticalrelease.pdf https://digital.nhs.uk/data-and-information/data-collections-and-data-sets/data-collections/reference-costs

## Abbreviations

CEAC: cost-effectiveness acceptability curve
ESIP.H: Economics of Smoking in Pregnancy Household
ICER: Incremental cost-effectiveness ratio
NHS: National Health Services
PSA: Probabilistic Sensitivity Analysis
QALYs: Quality-adjusted life years
RSDP: Reduced smoking during pregnancy
SDP: Smoking during pregnancy
SSS: Stop Smoking Services
UK: United Kingdom
