# Supplementary material for: Supporting pregnant women not ready to quit smoking: an economic evaluation
Source: BMC Pregnancy Childbirth. 2022 Nov 23;22:865. doi: 10.1186/s12884-022-05150-8 (PMC9686103; doi:10.1186/s12884-022-05150-8)
Supplement: Supplementary file 1 — Additional file 1. [file 12884_2022_5150_MOESM1_ESM.docx]

# ADDITIONAL FILE

# Supporting pregnant women who are not ready to quit smoking: An economic evaluation

Tuba Saygın Avşar^1^, Louise Jackson^2^, Pelham Barton^2^, Matthew Jones^3^, Hugh McLeod^4,5^

^1^ NIHR ARC North Thames and UCLPartners Academic Health Science Partnership, Department of Applied Health Research, University College London, UK

^2^ Health Economics Unit, University of Birmingham, UK

^3^Division of Primary Care, University of Nottingham, UK

^4^ Population Health Sciences, Bristol Medical School, University of Bristol, UK

^5^ The National Institute for Health Research Applied Research Collaboration West (NIHR ARC West) at University Hospitals Bristol and Weston NHS Foundation Trust, UK

**Correspondence to:**

Tuba Saygın Avşar, Department of Applied Health Research, University College London, WC1E 7HB. Email: [t.avsar@ucl.ac.uk](mailto:t.avsar@ucl.ac.uk)

**Declarations of competing interest:** None

**Funding:** Tuba Saygın Avşar’s time is supported by the National Institute for Health Research Applied Research Collaboration (NIHR ARC) North Thames and UCLPartners. Hugh McLeod’s time is supported by the NIHR ARC West. The views expressed in this study are those of the authors and not necessarily those of the NIHR or UCLPartners.

**Data statement:** Not applicable.

Table A1: Unit costs used to estimate the intervention costs

| Cost area | Unit cost (2017/18) | Source |
| --- | --- | --- |
| First counselling session including training | £45.79* | SNAP trial ^1^ |
| Antenatal community midwife visit | £56.14 | NHS Reference Costs ^2^ |
| Postnatal community midwife visit | £67.81 | NHS Reference Costs ^2^ |
| CO monitor | £0.56* | SNAP trial ^1^ |
| Printing | £1.21* | SNAP trial ^1^ |
| Voucher per specimen | £40 | Based on the study by Boyd et al. ^3^ |
| Initial voucher | £40 | Based on the study by Tappin et al. ^3^ |
| Postage costs for vouchers | £1.8 | Royal mail signed first class delivery price ^4^ |
| *Inflated to 2017-2018 prices based on national inflation indices ^5^ | | |

**DETERMINISTIC SENSITIVTY ANALYSIS**

Postpartum quit rates used in the base-case are shown in Table 2. A detailed description of how these figures were estimated is provided elsewhere^6^. They were reduced by half in the sensitivity analysis.

Table A2: Postpartum quit rates amongst mothers in the base-case

| Partner smokes | Yes | No |
| --- | --- | --- |
| LS | 0.17 | 0.23 |
| MS | 0.08 | 0.13 |
| HS | 0.06 | 0.10 |

**Figure A1: Deterministic sensitivity analysis for the base-case**

**Figure2: Deterministic sensitivity analysis for the cautious-case**

Table A3: ESIP.H outcomes of the cautious-case analysis

|  |  | Deterministic |  | Control (PSA) | | | Intervention (PSA) | | | Incremental outcomes (PSA) | | |
| --- | --- | --- | --- | --- | --- | --- | --- | --- | --- | --- | --- | --- |
| End of pregnancy | **Control** | **Intervention** | **Incremental** | **Mean** | **95% CI** | | **Mean** | **95% CI** | | **Mean** | **95% CI** |  |
| Number of reducers | 0 | 286 | 286 | 0 | 0 | 0 | 285 | 250 | 322 | 285 | 250 | 322 |
| Number of infants lost | 105 | 96 | -9 | 105 | 103 | 108 | 96 | 95 | 98 | -9 | -10 | -8 |
| Number of infants born prematurely | 70 | 70 | 0 | 70 | 69 | 71 | 70 | 69 | 70 | 0 | 0 | 0 |
| Number of infants born with LBW | 103 | 97 | -6 | 104 | 102 | 105 | 97 | 96 | 98 | -6 | -7 | -5 |
| Expected LYs per mother | 0.71 | 0.71 | 0.0047 | 0.71 | 0.71 | 0.71 | 0.71 | 0.71 | 0.71 | 0.005 | 0.004 | 0.006 |
| Expected QALYs per mother | 0.63 | 0.64 | 0.0097 | 0.63 | 0.62 | 0.64 | 0.64 | 0.63 | 0.65 | 0.010 | 0.006 | 0.014 |
| Incremental cost per mother and offspring | £7,050 | £7,710 | £660 | £6,393 | £3,457 | £11,529 | £7,047 | £4,067 | £12,229 | £654 | £533 | £806 |
| Incremental cost per QALY |  |  | **£68,150** |  |  |  |  |  |  |  |  | **£65,281** |
| End of one year after delivery |  |  |  |  |  | |  | | |  |  |  |
| Number of quitters (mother) | 127 | 156 | 30 | 127 | 0 | 0 | 0 | 0 | 0 | 0 | 0 | 0 |
| Expected LYs per mother and infant | 2.53 | 2.55 | 0.0134 | 2.53 | 2.53 | 2.54 | 2.55 | 2.54 | 2.55 | 0.013 | 0.012 | 0.016 |
| Expected QALYs per mother and infant | 2.34 | 2.36 | 0.0273 | 2.34 | 2.30 | 2.37 | 2.36 | 2.33 | 2.39 | 0.027 | 0.018 | 0.038 |
| Expected cost per mother and infant | £7,463 | £8,093 | £630 | £6,801 | £3,855 | £11,904 | £7,403 | £4,436 | £12,609 | £602 | £478 | £748 |
| Incremental cost per QALY |  |  | **£23,051** |  |  |  |  |  |  |  |  | **£22,053** |
| Offspring age 15 (end of childhood) |  |  |  |  |  | |  |  | |  |  | |
| Expected LYs per child | 10.25 | 10.35 | 0.1038 | 10.25 | 10.21 | 10.27 | 10.35 | 10.33 | 10.37 | 0.104 | 0.090 | 0.123 |
| Expected QALYs per child | 10.01 | 10.13 | 0.1234 | 10.02 | 9.68 | 10.21 | 10.14 | 9.84 | 10.31 | 0.124 | 0.101 | 0.165 |
| Expected cost per child | £7,272 | £7,581 | £309 | £7,109 | £4,379 | £12,476 | £7,399 | £4,694 | £12,784 | £289 | £84 | £475 |
| Incremental cost per additional QALY |  |  | **£2,505** |  |  |  |  |  |  |  |  | **£2,338** |
| Mother and offspring lifetime combined |  |  |  |  |  | |  |  | |  |  | |
| Expected LYs | 49.23 | 49.49 | 0.2562 | 49.26 | 49.13 | 49.39 | 49.51 | 49.40 | 49.65 | 0.256 | 0.223 | 0.301 |
| Expected QALYs | 44.06 | 44.36 | 0.3051 | 44.24 | 43.52 | 44.84 | 44.54 | 43.82 | 45.12 | 0.304 | 0.254 | 0.371 |
| Expected cost | £37,639 | £37,993 | £355 | £35,930 | £32,092 | £41,292 | £36,260 | £32,373 | £41,763 | £331 | £116 | £527 |
| Incremental cost per reducer at delivery |  |  | £1.24 |  |  |  |  |  |  |  |  | £1.16 |
| Incremental cost per QALY |  |  | **£1,162** |  |  |  |  |  |  |  |  | **£1,086** |
| Household (mother, partner, and offspring) |  |  |  |  |  | |  |  | |  |  | |
| Number of reducers at delivery | 0 | 286 | 286 | 0 | 0 | 0 | 285 | 250 | 322 | 285 | 250 | 322 |
| Expected LYs | 74.1426 | 74.4013 | 0.2587 | 74.20 | 74.00 | 74.43 | 74.46 | 74.27 | 74.68 | 0.259 | 0.225 | 0.303 |
| Expected QALYs | 66.4124 | 66.7193 | 0.3068 | 66.59 | 65.44 | 67.50 | 66.90 | 65.77 | 67.78 | 0.306 | 0.255 | 0.374 |
| Expected cost | £70,932 | £71,230 | £298 | £68,469 | £62,593 | £75,028 | £68,740 | £62,788 | £75,265 | £271 | £46 | £476 |
| Incremental cost per reducer at delivery |  |  | £1.04 |  |  |  |  |  |  |  |  | £0.95 |
| Incremental cost per QALY |  |  | **£970** |  |  |  |  |  |  |  |  | **£1,045** |
| Return on investment |  |  |  |  |  |  |  |  |  |  |  |  |
| ROI1 one year after delivery |  |  | -0.04 |  |  |  |  |  |  | -0.08 | -0.17 | -0.03 |
| ROI1 offspring and mother lifetime |  |  | 0.42 |  |  |  |  |  |  | 0.07 | 0.02 | 0.13 |
| ROI1 household lifetime |  |  | 0.51 |  |  |  |  |  |  | 0.43 | 0.18 | 0.75 |
| ROI2 one year after delivery |  |  | 1.31 |  |  |  |  |  |  | 0.44 | 0.25 | 0.67 |
| ROI2 offspring and mother lifetime |  |  | 15.49 |  |  |  |  |  |  | 2.63 | 0.73 | 0.73 |
| ROI2 household lifetime |  |  | 15.67 |  |  |  |  |  |  | 15.57 | 11.83 | 20.39 |

**References**

1. Cooper S, Lewis S, Thornton JG, et al. The SNAP trial: a randomised placebo-controlled trial of nicotine replacement therapy in pregnancy - clinical effectiveness and safety until 2 years after delivery, with economic evaluation. *Health Technology Assessment.* 2014;18(54).

2. Department of Health. *NHS Reference Costs: Financial year 2017-18.* London2018.

3. Tappin D, Bauld L, Purves D, et al. Financial incentives for smoking cessation in pregnancy: randomised controlled trial. *BMJ-British Medical Journal.* 2015;350.

4. Royal Mail. Your handy guide to our UK and International parcel and letter service prices: Valid from March 2017. <https://www.royalmail.com/sites/default/files/Royal-Mail-Mailmark-Franking-Wallchart-March-2017.pdf>. Published 2017. Accessed 07.09.2020.

5. Curtis L, Burns A. *Unit Costs of Health and Social Care 2018, Personal Social Services Research Unit.* Canterbury: University of Kent;2018.

6. Saygın Avşar T. *Economic Evaluation of Smoking Cessation Interventions for Pregnant Women*. Birmingham, UK: Health Economics Unit, University of Birmingham; 2020.
